# *Observations and Experiments Made with an Intention to Employ* Galvanism *in the Cure of Several Diseases*

**Published:** 1803-02-01

**Authors:** 

**Affiliations:** Berlin


### *Observations and Experiments made with an Intention to employ* GALVANISM *in the Cure of several Diseases, by* DR. GRAPENGIESSER, *of Berlin*; *communicated by* DR. NOEHDEN.

[ Continued from Vol. VIII. p. 324. ]

### *Method of applying Galvanism in Cases of Weakness of Sight and Amaurosis.*

IN order to excite the paralytic optic nerve, I endeavour to irritate the three branches of the fifth pair, by means of Galvanism; with respect to these two phenomena, I strongly suspect that a connexion subsists between them and the above nerve, though no anatomical proof can be adduced for it. The phenomena I allude to, are, first, the sternutation, which is frequently occasioned on suddenly stepping from the dark into sun-light; and, secondly, that species of amaurosis, which supervenes after a wound on the eye-brows, in the region of the frontal nerve. I therefore chiefly directed my attention to the nervus frontalis and naso-ciliaris, from which those phenomena proceed. To this end I procured a small silver probe, made of the size represented in tab. 1, fig. 6, by which I could touch as much of the surface of the nerves of membrana pituitaria nasi as possible. This probe was fastened to the silver side of the battery, by means of a silver chain, and another conductor, fig. 7, to the zinc side by a brass chain. The silver probe is put into the nose by the patient himself, while the operator touches the region of the frontal nerve, after it has been previously moistened with the knob (*b*) of the brass director, fig. 7, either without interruption or at momentary intervals.

In cases where a stronger irritation is intended, a small blister should be previously applied, then this method requires a less number of strata. The nose becomes gradually excoriated by the entrance of the Galvanic fluid, which renders its application extremely painful, so as hardly to be borne by the patient. In this case, the silver probe may be applied to the upper jaw above the upper molar teeth, in the inside of the mouth. But this application, when often repeated, excites a violent degree of the tooth-ach in several patients, which obliges us either to return to the first method, or to apply the silver probe externally on the cheeks, that require to be previously moistened. It is in general advisable to change, from time to to time, the place where the Galvanism is to be applied. The most efficacious irritation and shock ensues, however, on touching the cornea with the small knob (*a*) of the brass conductor, fig. 7.

The next effect of these different modes of application is a more or less intense appearance of lightning, depending on the degree of incitability which the optic nerve possesses, and on the efficacy or power of the machine; immediately after a painful sensation and an increased secretion of humours in the glandulæ lacrymales and meibomianae is perceived. The method of application, which was last mentioned, occasions the appearance of lightning in a most intense degree; and even in that case, when no lightning could be excited by the other modes of application. The shock is, at the same time, so penetrating, and the pain so acute, that the patient will hardly suffer the application from more than 20 strata, as a greater number generally occasions a redness and inflammation of the eyes, which generally lasts for several hours, and sometimes two or three days. This method, however, has generally proved very powerful and efficacious, even in cases where the optic nerve was paralysed to such a degree, that not the least appearance of lightning could be excited by the other methods; yet here it had the intended effect, and sometimes the power of perceiving the lightning, on touching the frontal nerve, was restored by it. It may be asked, whence that violent shock of the optic nerve proceeds, on touching the cornea, this membrane being endued with few or no nerves at all? But it is very probable, that the Galvanic fluid passes through the cornea, as being a moist membrane, and is conducted through the humours of the eye (humores oculi) immediately to the optic nerve.

If the disease is curable by means of Galvanism, the iris becomes first moveable, while the sight increases, in case the paralysis of the optic nerve should be attended by that of the iris. Sometimes I have observed the pupil to become moveable without its having any favourable effect on the sight. The sight is gradually restored in the following manner: Every thing about the patient, the ground and atmosphere, appear to him as if covered with snow; and as the sight increases, he imagines that the sun shines on that snow. The objects begin now to appear clearer and more distinct, the patient distinguishes colours, and thus advances towards recovery.

*Method*

### *Method of applying Galvanism in Diseases of the Auditory Organ.*

For applying Galvanism in diseases of the auditory organs, I have hitherto used five different methods, the first of which consists,

1. *In acting on both Ears at the same Time.*—For this purpose, the two conductors, tab. 1, fig. 3, which I found to be the most convenient and useful, were fastened to the two conducting plates, tab. 2, fig. 2, *b*. and *c*. The conductors consist of a silver wire, bent at one end exactly in the direction of the meatus auditorius, and terminating in a small knob, which must be wrapped in linen. They pass through a glass tube, which is likewise provided with a globe, for preventing its entering too deep into the meatus auditorius by any motion of the patient. The whole instrument should exactly correspond with the largeness of the auditory canal; at least, the knob must entirely fill it. If the knob is not covered with linen, and the conductor brought in without its glass sheath, the ear will be immediately excoriated; a circumstance which renders the farther application, at least for some time, impossible; and before I found out the above expedient, the continuation of my experiments was frequently interrupted, as the patient could not bear the pains caused by the uncovered conductor, in the sore meatus auditorius, and some of them fell into a lipothymy. The ends of the conductors being covered with linen, and moistened, are brought into the meatus auditorius, that has also been moistened; from thence the Galvanic stream, running through the nerves of the meatus with great rapidity, is communicated to the auditory nerve, and immediately occasions in some patients the most violent sounding and tinkling in the ears; and the optic nerve being likewise affected, considerable lightnings appear before the eyes; several patients, likewise, complain of a giddiness, soon after the application, which however is only of short duration.

The degree of irritation, produced by the Galvanic battery, as well as the period during which it is to be applied, must be adapted to the degree of incitability of the patient, and to the degree of the disease itself; a circumstance, which depends entirely on the judgment of the physician. I generally suffer the action of the battery to continue for five minutes, sometimes ten minutes, in several patients fifteen, and sometimes even for half an hour, which is once or twice repeated in a day. It frequently happens, that the Galvanic stream is sometimes very

 weak, weak, but, at other times, it acts too violently, which however may be easily prevented by shaking the chains from time to time. It has been already mentioned, that the conductor which is connected with the zinc side, produces a greater effect than that at the silver side, on which account it is advisable to change the conductors from time to time, when both ears are equally affected; but when one ear is worse than the other, the conductor of the zinc side should be applied to the ear that suffers the greatest degree of deafness. For better convenience in the application of the two conductors, the machine, tab. 2, fig. 1, has been invented; by means of which the conductors tab. 1, fig. 3, are kept in proper order and direction, without the assistance of a third person.

2. *Method of applying Galvanism on one Ear.*—This is done in three diffeient ways. 1. By bringing the director, tab. 1, fig. 5, into the meatus auditorius, while the patient touches with a silver spoon, which he holds in his moist hand of the same side, the other end of the pile, by which means the following chain is formed: Zinc pole, ear, neck, arm, silver pole; and vice versa. But this method produces the least immediate action on the organ of hearing. 2. The conductor, tab. 1, fig. 3, being brought into the meatus auditorius, I touch the processus mastoideus, on which the skin has been previously moistened with a silver spatula, connected with the other end of the pile. By this mode of application, the Galvanic stream passes through a part of the auditory organ, viz. the labyrinth. 3. The most poweful and useful method is undoubtedly the following: The conductor, tab. 1, fig. 3, is brought into the meatus auditorius, while the other, tab. 1, fig. 4, is applied to the tuba Eustachii in the mouth, at which moment the patient perceives a sensation similar to that of a series of small globules running through the ear. There cannot be a better method by which the Galvanic fluid may be immediately conducted to the auditory nerve, than this, notwithstanding its being attended with great inconveniencies both to the patient and the operator, as it frequently excites in the former an effort to vomit, which makes the operator desist from the further continuation of the experiment, and obliges him to remove the instrument. The conductor of which I make use for that experiment, consists of a silver wire six inches long, which ends in a small knob; it passes through a glass tube, which is bent at one end, for the purpose of applying it more conveniently behind the velum palati pendulum; it ought to be likewise wise provided with a globe below the knob of the wire, by which means the appetency to vomit is less excited. All these methods of applying Galvanism on one ear, of which the last mentioned is the most advantageous, act almost exclusively on the auditory organ, and only in an inconsiderable degree on the whole head; on which account they produce no giddiness, nor excite congestions of blood towards the head. The last method of applying the Galvanism on the affected organ of hearing is,

3. *The Application of the simple Galvanic Chain on Wounds, occasioned by Blisters behind the Ears.*—A blister being applied behind each ear, a plate of silver, and another of zinc, are put on the two places which have been deprived of their epidermis, after which they are connected by means of a gold and silver chain; the first of which appears to increase the efficacy of the apparatus. The whole is preserved in its proper situation by a head bandage. The apparatus shows itself particularly efficacious, when the zinc plate lies close to the place, while the silver plate is rather loosely fastened, that it may be alternately pressed to the wound, and removed from it. On pressing this plate close to the wound, the appearance of lightning and a gentle tinkling in the ear will be perceived. The time during which this apparatus is suffered to act, depends on the incitability of the patient, and similar circumstances. In general, I found eight to ten, and sometimes only five hours to be sufficient for producing some effect.

### *Method of applying Galvanism in the Paralysis of the Sphincter Vesicae Urinariae.*

The zinc conductor is brought into the intestinum rectum, while the conductor of the silver side is applied above the arcus ossium pubis, on the moistened skin, or on a wound made by a blister. In women, the first conductor may be immediately applied to the collum vesicae through the vagina.

### *Method of applying Galvanism in Chronical Hoarseness, Aphonia, White Swellings, Struma, Rheumatism, &c.*

It is in these diseases not only intended to excite, but likewise to act by Galvanism as a discutient and derivant. With this view, I make use as well of the simple Galvanism as also of that of the battery, either on the moist skin or on wounds made by blisters, as has been already described.

### Cases of Diseases, in which Galvanism has been employed.

### I. *Cases of Paralyses of the Extremities.*

Case 1. Mr. G. C. W. Z. 18 years of age, was on the 28th of February attacked with an indisposition and great weariness, on which account he went to bed soon after dinner, and continued to sleep till the next morning. Being with difficulty awakened by his servant, and finding himself very ill, a surgeon was sent for, who, on examining the patient, discovered that his left side was paralysed, and the shoulder, hip, and dorsum pedis livid; the arm and foot were quite unmoveable, and a slight motion was only left in the fingers. He was ordered to take febrifuges, after which the paralysis of the arm entirely disappeared. He began to take guaiacum, during the use of which the hip got slowly better. At the latter end of March, being called to attend him, I found him without fever, but the left leg was quite paralysed, and an inflammation had taken place on the left foot, which was attended with violent pains. I took the whole for a remainder of a febris rheumatica, apoplectica epidemica, (which at that time was very common in Berlin,) and the inflammation to be, accordingly, of a rheumatic nature. With this view I prescribed powders of calomel, sulphur. antimonii auratum, camphor and opium, to be used with an infus summitat. arnicæ. Some time after I gave him a solution of corrosive sublimate with camphor and opium, and a decoction of lign. guaiac. dulcamara, serpentaria, arnica; which I changed soon after with pills of guaiac. extr. arnicae, alcal. miner. caustic. sulphur and squills; by which remedies the inflammation and pains were removed in the course of about a month. The patient being, however, considerably weakened, I ordered him to take bark with rad. valerian, and summitat. arnicae, which had however no effect on the paralysed feet. Having therefore discontinued the bark, I determined to apply the Galvanism. Two batteries were accordingly raised, one of copper and zinc plates, the other of silver and zinc plates, each of 50 strata, from which, by means of two conductors, as tab. 1, fig. 7, the Galvanism was led to two places, one on the outside of the knee, the other on the back of the foot, above the toes, both which had been previously excoriated by two blisters. This was done twice a day, and continued during one quarter of an hour. The patient perceived by degrees more power and sensation in the whole foot, so as to be able to move and tread upon it, and and he even began to walk along some chairs by which he supported himself. A fortnight after he was able to move the foot up and down, though still with great effort; its strength, however, daily increased, and on the 16th of June, when he discontinued the application of Galvanism, and was preparing to go to the baths of Toplitz in Bohemia, he had so far recovered the use of his feet, that he could walk with much ease, and stretch his foot as well as before, though the contraction of it was not yet entirely re-established.

Case 2. Charlotte ———, aged 26, had till her 22d year enjoyed the best health; but about this period being afflicted with great vexation and sorrow, she was frequently attacked with head ach, which she particularly suffered in the morning; her appetite was much diminished; and one day she felt a great difficulty in speaking, when conversing with some persons that came to see her. These complaints were the forerunners of an apopletic fit, which soon after supervened. She was entirely deprived of her senses, and quite speechless; the right sight was completely paralysed. Being treated for some time by an intelligent physican, the use of her senses was restored to her, but the speechlessness and the paralysis of the right side remained. She used afterwards, during a period of four years, the mineral baths at Freienwalde, Lauchstadt, Toplitz and Tarandt, by which the aphonia and the paralysis of the foot were removed, though the arm remained as lame as before. This was about the time when I first saw the patient, who was paralysed to such a degree, that she could hardly lift it up. The elbow joint was bent by a spastic contraction of the muscles, and likewise the fingers of the same hand were so contracted, that no power was able to open it. Whenever the Galvanism was applied, this stiffness of the elbow and of the fingers disappeared in a moment, and she could with ease stretch the fingers and the arm. But in order to produce this effect, a battery of 100 strata was required, by the application of which the affected parts became moveable; and after its being continued for a short time, the patient was enabled to lift the arm up to the head and to open the hand, though not so well as before.

Case 3. G. S, a taylor, 35 years of age, much addicted to the use of spirituous liquors, perceived about two years ago, one morning after having taken a glass of brandy, a sense of itching on the right side of his face, and a tetanic tetanic stiffness in the masticatory muscles, so that he could but half open his mouth. Some days after he felt the same sensation in his right foot, then gradually in his left and right hand. All these members were quite benumbed, stiff and lame; the patient could seize and hold large bodies, but with great difficulty; small bodies however, for instance, his needle, he was not at all able to manage. In this state he had continued for more than two years, notwithstanding all the medicines he used during that time; when I tried to apply the Galvanism of the battery against the paralysis of the arms, by letting him with his wet hands hold two silver spoons, with which he was ordered to touch from time to time, the two poles of a pile of 50 strata. This being continued for some days, the patient got not in the least better; on the contrary, the paralysis and immobility of the arms seemed to increase considerably, on which account I desisted from any farther application.

II. *Cases of Amaurosis and Weakness of Sight.*

Case 1. D. G. a merchant of Berlin, aged 40, had received in his childhood, after the small-pox, a cataracta partialis on the left eye, which hindered him from seeing the objects with that eye as distinctly as with the other. About four years ago, however, he had the misfortune to be affected, after an acute fever, with an imperfect amaurosis on the right eye, as he could only see sufficient to be able to walk in the streets, and to perceive when persons approached towards him, though it was impossible for him to distinguish their physiognomy and the colour of their dress. This complaint frequently changed, as it sometimes got better, and sometimes worse; when it had become very bad, he was prevented from leaving his apartments, all objects appearing black to him, and he was in danger of runing against the persons that met him. When his complaint was in the best state, he could distinguish small pieces of money, but could neither write nor read. This periodical increase and decrease of the fever he was inclined to ascribe to the changes of the moon. When he consulted me, about two years ago, he seemed to be extremely debilitated; on which account I prescribed to him bark, valerian, arnica, camphor, by the use of which he got considerably better, so that he could even distinguish colours and small coins. On discontinuing, however, these remedies, he relapsed into the former state. He afterwards underwent another treatment, but without much benefit; and the only thing that seemed to have a favourable effect, was a large blister on the the back, kept in a state of suppuration. It was about this time that I begun my experiments with Galvanism, and I therefore offered to employ this stimulus for the cure of his affection, to which he readily agreed. Galvanism was accordingly applied, by bringing alternately one conductor into the nose or mouth, and by touching with the other the excoriated region of the frontal nerve. During this application of Galvanism, the patient made such astonishing progress towards recovery, that after a period of about 18 days, he was enabled to read the Berlin newspapers, which he could never do before, and he thought himself so far cured, that he discontinued the farther application, and gave himself up to his former intemperance in spirits and in venery. Three weeks after his sight became almost as bad as ever; and though he had again recourse to Galvanism, in combination with internal exciting remedies, and a large blister behind the ears, the good effects of this repeated application manifested themselves but slowly; and as this seemed to be too tedious to him, he quitted me, and I do not know what is since become of him.

Case 2. Miss R. I. had been afflicted for about 15 years with a dimness of sight, or scotomy on both eyes, of which no other cause could be found out, but that the patient, about a year before the accession of this complaint, being much heated with dancing, had gone into the moist air, and caught cold; besides this, she had much used her eyes by reading and working by candle light. She took for several months pills of soap, tartar emetic and millepedes, but without avail. The sight gradually decreased on the left eye, till a perfect cataract was perceived, which being extremely large, and the pupil immoveable, was thought to be complicated with amaurosis. Notwithstanding these unfavourable circumstances, she underwent the operation; which was, however, so successfully performed on her, that she could distinguish large objects and the persons that were about her; ten days after the operation, she complained of a pressure and pains in the eye, which were found to have arisen from a prolapsus iridis, that was soon successfully removed by gentle caustics. The patient could pretty well distinguish large objects, though there always remained a dimness of sight, for which she was ordered to use the strongest exciting remedies, electricity, &c. by which, however, the complaint increased, and a very bad cataract appeared also on the right eye. After its being operated with great difficulty, a violent inflammation inflammation and suppuration supervened, by which the eye was lost. About four years ago the sight of the left eye entirely disappeared, and different remedies were in vain applied, amongst others belladonna, which she took in very large doses, without experiencing any good effect on the sight. At last she determined to use the Galvanism, which being applied in the manner above described, produced not the least appearance of lightning in the eye; on which account I intended to discontinue the experiments, thinking at that time the farther application of this stimulus quite useless, if it did no longer effect the appearance of lightning in the eye. I was, however, prevailed by the attending physician to continue my experiments, as he intimated to me, that the nature of that powerful stimulus was not yet so sufficiently known, as to be able to fix the limits of its efficacy. The application of Galvanism being accordingly continued, the patient began to perceive the lightning, particularly on the conductor being applied to the cornea. This mode of application made the pupil, which had been before quite immoveable, so irritable, that it became considerably contracted in sun light. But whenever a considerable shock was given to the eye, an inflammation supervened, which obliged us to discontinue the experiment for several days; a circumstance, which made the use of this stimulus insufficient for producing any farther effect, in curing the disease against which it was applied.

Case 3. D. M. a silk weaver, thirty-four years of age, requested my assistance in a dimness of sight, with which he had been affected for several years. I found that he had an imperfect amaurosis, which let him distinguish some objects; for instance, the fingers, but he was unable to distinguish the colours. On enquiring into the cause of this affection, I thought it had originated from excess in drinking and in venery, on which account the application of Galvanism seemed to be indicated. It was accordingly applied, in combination with other *excitantia* and *roborantia,* as peruvian bark, valerian, arnica, &c. by which the sight of the patient was thus far restored, that he could perceive the difference of colours, if he looked close on them; the cloud spread before his eyes had entirely disappeared, and I had the satisfaction to find that he could again do his business as before.

Case 4. I. Valenti, eighteen years of age, a native of Poland, had been afflicted about nine years ago with an inflammation of the eyes, after the incautious extirpation of a plica polonica. Some years after, a dimness of sight supervened, which terminated in a complete amaurosis on both eyes. This affection had already lasted during seven years, when he applied to me for assistance. Having administered to him different remedies, particularly mercurials, I determined at last to have recourse to the application of Galvanism, which I chiefly conducted to the cornea; at the same time he was ordered to take powders of camphor, valerian, and arnica. After having continued this method of cure for about six weeks, the patient had so far recovered his sight, that he could distinguish large objects, if brought close to his eyes. The structure of his eyes, however, being naturally such as to dispose him to myopia, I have but little hopes that his sight will ever be perfectly restored.

Case 5. I. K. forty-five years of age, had an amaurosis on both eyes, and he could only with the left eye distinguish light and darkness. It had been preceded by a scotomy and diplopia, and was most probably occasioned by using the eyes too much by candle-light, and by being continually occupied with writing. He complained at the same time of an hemicrania. Several remedies, as purgatives, emetics, a seton, blisters, having been used without much benefit, I resolved to apply Galvanism on the left eye; but at the same time I ordered him to take the succus expressus millepedum, with powders of camphor, arnica, and valerian. Galvanism being applied for about eight days, the patient perceived some favourable change; he was able to see the flame of a lamp, and to distinguish large black letters, that were written on a white wall. The eye gradually improved in its appearance, it recovered the natural gloss and vivacity, and sometime after the patient could distinguish persons and even the outlines of their physiognomy, and other large objects. A stronger shock being accidentally given to the eye, the sight got worse again, which however was soon remedied by a more cautious application. This patient continues at present this method of cure, from which he has every reason to expect farther success.

Case 6. I. H. K. aged fourteen years, was born, like all his brothers and sisters, with an imperfect amaurosis; he could could distinguish the outlines of a person, the number of horses that were put to a carriage, and other large objects; but there were different forms, fiery crowns, and other phantasmata continually swimming before his eyes, which lay deep in the orbits, and were continually in a trembling motion. The pupil was very little moveable. I tried Galvanism on this unhappy boy, in combination with other inciting remedies; but all my pains and repeated experiments were ineffectual, whence I concluded, that there might most probably be an organic and local fault in the eyes, which must consequently have defeated any method of cure.

Case 7. M. W. a girl, fourteen years of age, complained for some years of so great a weakness of sight, that it was impossible for her to read one page of a book without suffering pain, and she was hindered from reading any farther by a flood of tears that issued from both eyes. After having used several strengthening eye waters without avail, she was perfectly cured of that complaint by an application of Galvanism continued during two months.

### III. *Cases of Deafness and Difficulty of Hearing.*

Case 1. H. S. a boy, twelve years of age, had from his earliest childhood been deaf to such a degree, that notwithstanding his natural vavicity and faculties, he had, as yet, remained incapable of speech. It could not be ascertained whether he was born deaf and dumb, or whether his deafness had arisen from a cold draught of air to which he had been for some time exposed, when quite young. In the seventh year of his age he received the small-pox, after which it was perceived that he heard a little; at least, his attention was drawn towards noise and sounds; which seemed to prove that the cause did not originate in a deficient structure of the organ of hearing, but rather in the nervous system. He was educated in the Institution for Deaf and Dumb, at Berlin, under the direction of the celebrated Professor Eschke, who during his stay there observed, that he was not utterly deprived of hearing, but that, particularly at some periods, he had a sensation of strong sounds and loud noise. Under these circumstances, I thought myself entitled to expect some success from the application of Galvanism; and after the fourth time of its being applied, the boy, of his own account, made a sign that he could hear the noise of a carriage moving along, the the opening of a door, &c. The application of Galvanism having been repeated eight times, he could distinguish sounds that were less loud, and his faculty of hearing was gradually so much improved, that he could distinctly hear the beating of a watch. By the continued application of Galvanism, he perfectly recovered his sense of hearing on one ear, and he is, at present, able to repeat word by word what is said to him.

Case 2. A lady having been affected with an otitis rheumatica, experienced on both ears such a difficulty of hearing, that she could not understand what was spoken at a little distance; particularly when several persons conversed with one another, she was incapable of comprehending what was said. As she had already used a great number of remedies without any avail, except the sea baths, which seemed to do some good, I resolved to try Galvanism on both ears. The patient became a little giddy from each experiment, and perceived a sounding in the ears, which she compared to distant thunder. After the experiment being over, she felt herself very easy in the head, the tinkling in the ears, with which her complaint was attended, had considerably decreased, and she perceived, after the third experiment, that she could hear much better than before. The patient, however, was prevented from continuing the application of Galvanism, by her subsequent departure from Berlin.

Case 3. H. B. aged forty-seven, had been quite deaf for above seven years, the cause of which he attributed to a severe cold. I applied the Galvanism, in this case, after all the five methods; and as the patient had so much perseverance as to continue the application for above four months, he thus far recovered his sense of hearing, that he could understand what was spoken to him slowly, distinctly, and not too loud. The remaining difficulty of hearing could not be removed by Galvanism, though the experiments were continued for some time.

Case 4. S. B. fifty-four years of age, had been deprived of his sense of hearing by a violent cold and a previous rheumatism, which being removed by proper remedies, a deafness remained, which, notwithstanding the best remedies, gradually increased to such a degree, that he could only understand what was said through a hearing trumpet. Galvanism was applied on him for about six weeks, during which time he began to hear better than before, but the singing in the ear did not cease, it rather seemed to increase. The favourable appearance of this cure, however, was of short duration, and in two months after he relapsed into his former state.

Case 5. L. T. a girl, twenty-two years of age, had been affected with a difficulty of hearing for several years, which was attended with a tinkling in the ears. This complaint was hereditary in the family, but had been increased with her by the use of the waters of Toplitz and Carlsbad in Bohemia. Galvanism proved in this case very efficacious the first fortnight, as the sounding in the ears disappeared with almost a perfect recovery of the sense of hearing. On continuing however the experiments during the period of fluxus mensium, of the entrance of which she had not informed me, the sounding in the ears came on again, and at the same time, the difficulty of hearing. Venæsection, foot baths, and cooling remedies being applied without avail, recourse was had to Galvanism, but it had not the desired effect this second time.

Case 6. W. A. aged nineteen, had a difficulty of hearing, attended with a very disagreeable tinkling in the ears, with which she had been afflicted soon after having had the small-pox. As I discovered several symptoms of scrophula, I ordered her to take the proper remedies, as calomel, sulphur antimonii auratum, cicuta, a decoction of lign. guaiac. radix. caric. arenariae, stipit. dulcamar. rad. halenii, &c. and at last the Peruvian bark, by which the scrophulous symptoms disappeared, but the difficulty of hearing remained as before. I now applied the Galvanism of the battery on both ears; and at different times, the simple Galvanic chain on wounds behind the ears, made by blisters. After having used this method of cure during six weeks, her sense of hearing was perfectly restored, and the tinkling in the ears entirely disappeared.

Case 7. Mr. H. P. M. had been frequently affected with cardialgies, preceded by a vertigo and sounding in the right ear, which the first time of his complaint disappeared, together with every fit of cardialgia. Some time after, however, the sounding remained for a little while, when the fit of cardialgia was over, and frequently returned without it, till at last it took a fixed seat in that ear. By degrees it was attended with deafness, which remained after after the cardialgia had disappeared of itself. Galvanism was applied on this ear by conducting the Galvanic stream through the tuba Eustachii, and the meatus auditorius externus. The patient perceived a favourable effect after the repeated application, the sounding in the ears decreasing gradually, and the faculty of hearing being improved. He could hear the beating of a watch, and understood what persons spoke at some distance from him. A cold prevented him from continuing the experiments.

Case 8. A. C. a girl, twenty-one years of age, of a scrophulous habit, had been affected for above twelve years with a difficulty of hearing, and a tinkling in the ears. On taking different anti-scrophulous remedies, as calomel, bark, &c. she was freed of the latter complaint; and for the deafness, I applied the Galvanism of the battery, and the simple Galvanic chain in the usual way, by which means she was perfectly cured.

Case 9. A boy, five years of age, born deaf and dumb, was galvanised in the above described manner, which having been continued for about a fortnight, had such an effect that he could hear strong sounds, and noise to which he had never before paid any attention. It is probable that this boy would have made more progress towards the recovery of his sense of hearing, had I not been obliged to discontinue my experiments; a circumstance which likewise took place with another child, born deaf and dumb, in which the application of Galvanism seemed to promise a similar success.

These are all the cases stated by Dr. Grapengiesser, in which he had himself employed, with more or less success, the Galvanic power. At the end of his work he has subjoined a few observations relative to the application of Galvanism, made by Mr. Volker, who succeeded in curing a chronical rheumatism by the means of Galvanism, and by Dr. Flies, who relates two cases of paralysis, in which the Galvanic agent proved efficacious, but particularly a case of amâurosis incipiens, which was perfectly cured by the timely and proper application of this powerful stimulus.

